# Functional Roles of Calreticulin in Cancer Biology

**DOI:** 10.1155/2015/526524

**Published:** 2015-03-31

**Authors:** Yi-Chien Lu, Wen-Chin Weng, Hsinyu Lee

**Affiliations:** ^1^Department of Life Science, National Taiwan University, Taipei 106, Taiwan; ^2^Department of Radiology, Wan Fang Hospital, Taipei Medical University, Taipei 11696, Taiwan; ^3^Department of Radiology, School of Medicine, College of Medicine, Taipei Medical University, Taipei 11031, Taiwan; ^4^Department of Pediatrics, National Taiwan University Hospital and National Taiwan University College of Medicine, Taipei 100, Taiwan; ^5^Research Center for Developmental Biology and Regenerative Medicine, National Taiwan University, Taipei 116, Taiwan; ^6^Center for Biotechnology, National Taiwan University, Taipei 116, Taiwan; ^7^Angiogenesis Research Center, National Taiwan University, Taipei 116, Taiwan

## Abstract

Calreticulin is a highly conserved endoplasmic reticulum chaperone protein which participates in various cellular processes. It was first identified as a Ca^2+^-binding protein in 1974. Accumulated evidences indicate that calreticulin has great impacts for the development of different cancers and the effect of calreticulin on tumor formation and progression may depend on cell types and clinical stages. Cell surface calreticulin is considered as an “eat-me” signal and promotes phagocytic uptake of cancer cells by immune system. Moreover, several reports reveal that manipulation of calreticulin levels profoundly affects cancer cell proliferation and angiogenesis as well as differentiation. In addition to immunogenicity and tumorigenesis, interactions between calreticulin and integrins have been described during cell adhesion, which is an essential process for cancer metastasis. Integrins are heterodimeric transmembrane receptors which connect extracellular matrix and intracellular cytoskeleton and trigger inside-out or outside-in signaling transduction. More and more evidences reveal that proteins binding to integrins might affect integrin-cytoskeleton interaction and therefore influence ability of cell adhesion. Here, we reviewed the biological roles of calreticulin and summarized the potential mechanisms of calreticulin in regulating mRNA stability and therefore contributed to cancer metastasis.

## 1. Structural Information of Calreticulin

**Figure 1:**
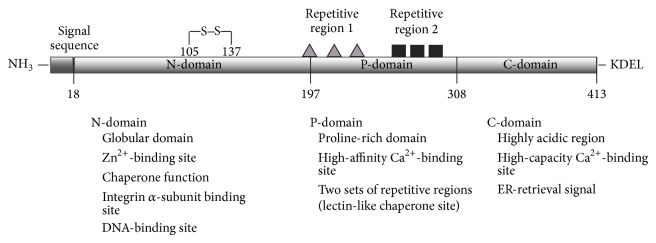
The protein structure and putative functions of calreticulin domains. The figure represents a schema of calreticulin. The protein contains three functional domains: N-domain, P-domain, and C-domain. There is a signal sequence at N-terminal and a KDEL ER retrieval peptide at C-terminal. The two sets of repeated regions are indicated by triangles and squares, respectively. The putative functions of each domain as shown.

Calreticulin (CRT) is a 46 KDa multifunctional protein predominantly located in endoplasmic reticulum (ER) and highly conserved in diverse species. It is synthesized with a cleavable signal sequence at N-terminal and an ER KDEL (Lys-Asp-Glu-Leu) retrieval signal at C-terminal. Structural predictions of CRT demonstrated that the protein is composed of three domains, including N-domain, P-domain, and C-domain (Figure 1) [1].

The N-terminal region of CRT is a globular domain containing eight antiparallel *β*-strands [2]. This domain can interact with *α*-integrins [3] and DNA-binding site of steroid receptor [4]. The disulfide bond formed by cysteine residues in the N-domain may interact with P-domain to generate important chaperone function of calreticulin [5].

The proline-rich P-domain contains two sets of three repetitive regions [6]. These repeated amino acid sequences form the lectin-like chaperone structures which are responsible for protein-folding function of CRT. Moreover, the P-domain of CRT is also a high-affinity and low-capacity Ca^2+^-binding region [7, 8].

The C-domain of CRT is a highly acidic region which is important for Ca^2+^-buffering functions. It binds to Ca^2+^ with high capacity and low affinity manner [9]. It is known that Ca^2+^ binding to this region plays a critical role in the interaction with other chaperone proteins in ER [1, 10].

Since there is a KDEL sequence for retrieval in the ER at C-terminal of CRT, it is not surprising that this protein is highly enriched within the ER lumen. However, evidences demonstrated that CRT is also expressed in cytosol [11] and on cell surface [12]. It has been reported that the C-domain is important for CRT retrotranslocation from ER lumen to the cytosol [13]. Further study also indicates that this retrotranslocation process is triggered by ER Ca^2+^ depletion [14]. In addition, some studies have shown that cytoplasmic CRT may interact with the cytoplasmic tail of *α*-integrin through the KXGFFFKR sequences [3, 15–17]. Furthermore, cell surface CRT is associated with phagocytic uptake and immunogenicity of cells [18]. These results provide more evidences for CRT as a multifunctional protein which may participate in various physical and pathological events in cells.

## 2. Biological Functions of Calreticulin

Over the past years, CRT has been proposed to participate in various physiological and pathological processes in cells. The two major functions of CRT inside the ER are protein chaperoning and regulation of Ca^2+^ homeostasis. Furthermore, accumulated studies indicate that non-ER CRT also regulates important biological functions including cell adhesion, gene expression, and RNA stability.

## 3. Protein Chaperone

ER is an important organelle for synthesis, folding, and transportation of secretory proteins. These functions are carried out by molecular chaperones which facilitate correctly protein folding and assembly. CRT is one of the well-characterized lectin-like ER chaperons for many proteins [19–22]. Recent evidences indicated that CRT is involved in quality control process during protein synthesis, including integrins, surface receptors, and transporters [1].

## 4. Calcium Homeostasis

Ca^2+^ is mainly stored in ER lumen and is a universal signaling molecule affecting many developmental and cellular processes [23]. Numerous reports indicated that Ca^2+^-binding chaperones influence Ca^2+^ storage capacity in the ER lumen [24–27]. CRT is considered as an intracellular Ca^2+^ regulator since it contains two Ca^2+^-binding sites in the P-domain (high-affinity, low-capacity) and C-domain (low-affinity, high-capacity) [7, 9]. More than 50% of Ca^2+^ stored in ER lumen associates with CRT [9]. Therefore, higher levels of CRT may lead to increase intracellular Ca^2+^ storage [28, 29]. In contrast, CRT-deficient cells have a lower capacity for Ca^2+^ storage in the ER lumen [9]. The cardiac development in CRT-deficient mice is defective due to the impaired Ca^2+^ homeostasis of CRT [30, 31]. Besides, abnormal function of CRT also associated with adipocyte differentiation and Henle's loop adaptation under osmotic stress [32, 33]. These findings further support that CRT plays crucial roles during Ca^2+^ homeostasis.

## 5. Cell Adhesion

The concept that CRT might be involved in cell adhesion is based on the regulation of focal contact via multiple mechanisms [34, 35]. It is clear that the extracellular matrix (ECM) molecules are important for focal contact formation. Several studies elucidated that alteration of CRT levels affects cell adhesion on various ECM [36–38]. Papp et al. implicated that CRT plays a role in the control of cell adhesiveness through regulation of fibronectin expressions and matrix deposition. These effects are mediated via Ca^2+^-dependent effect of CRT on c-SRC activity [39]. In addition, previous studies revealed that CRT-mediated cell adhesion might be due to direct interaction between CRT and integrins by binding to the cytoplasmic KXGFFKR motif of the integrin *α*-subunit [3, 40, 41]. These studies provided evidences that CRT plays a critical role in cellular adhesiveness.

## 6. RNA Stability

In 2002, Nickenig et al. first indicated CRT as a novel mRNA binding protein that destabilizes type I angiotensin II receptor mRNA by binding to AU-rich region in 3′-UTR [42]. Moreover, Totary-Jain et al. reported that CRT also binds to specific element in 3′-UTR of glucose transporter-1 mRNA and destabilizes the mRNA under high-glucose conditions [58]. These results identified a new function of CRT which is being a trans-acting factor which regulates mRNA stability.

## 7. Regulation of CRT Expression

The human calreticulin gene (*CALR*) is located on chromosome 19p13.2 with nine exons. Calreticulin promoter region contains several binding sites for reputed transcription factors and many of these factors have been identified as important modulators of CRT expression including NKx2.5, MEF2C, COUP-TF1, GATA6, Evi-1, and PPAR factors [59]. In addition, calcium depletion and ER stress were shown to be important activators of* CALR* transcription [60]. Recently, studies have also revealed that nerve growth factor (NGF) can also upregulate CRT expression in both ovarian cells and neuronal differentiation [61, 62]. These results suggested an involvement of CRT expression in various biological and pathological processes.

## 8. Clinical Impacts of Calreticulin Expression in Different Cancers

The correlation between CRT expression levels and tumorigenesis has been extensively studied in various cancers and most reports have indicated that tumor tissues express significant higher levels of CRT compared to normal tissues [63]. These clinicopathological significances for CRT in different cancers are summarized in Table 1. Studies have demonstrated that the CRT expression levels were positively correlated with clinical stages and lymph node metastasis in gastric cancer [49] and breast cancer [45]. In addition, patients with higher CRT levels had a poor survival rate in pancreatic cancer and esophageal squamous cell carcinoma [44, 48]. Other studies also revealed CRT expression levels to be significantly upregulated in oral cancer [43], breast ductal carcinoma [46, 47], colorectal cancer [50], prostate cancer [54], and vaginal carcinoma [55]. Furthermore, CRT levels not only increase in bladder cancer tissues [51], and urinary CRT has also shown to be a useful biomarker for bladder urothelial cancer detection [52]. Kageyama et al. implicated that the concentration of urinary CRT has a tendency to increase in high grade tumors [53]. These results indicate that increased CRT expression might play a crucial role during cancer progression.

On the other hand, the roles of CRT in ovarian cancer progression are inconclusive. Compared with primary tumors and solid metastases, reduced CRT expression was observed in malignant effusions of high-grade ovarian carcinoma along disease progression [56]. Moreover, CRT expression levels in effusions may be associated with better response to chemotherapy while the survival was not related to CRT expression [56]. Furthermore, in neuroblastoma, increased CRT expression is found to be associated with better prognosis and differentiated histologies [57, 64]. Therefore, the impact of CRT on tumor formation and progression may depend on different cell types and clinical stages.

Very recently, mutations in calreticulin gene were detected in myeloproliferative neoplasms (MPN) [65, 66]. Most patients with MPN including polycythemia vera (PV), essential thrombocythemia (ET), and primary myelofibrosis (PMF) were found to have mutations in Janus kinase 2 gene (JAK2) [67, 68]. For the remaining patients, mutations in CRT gene were identified [65, 66]. These CRT mutations include 52 bp deletion and 5 bp insertion of certain base pairs, which leads to frameshift mutations [69]. Proteins encoded by mutated CRT gene lack the C-terminal KDEL domain; therefore they may affect normal Ca^2+^ binding and cell growth.

## 9. Membrane Calreticulin as a Phagocytic Signal

An important role for CRT exposed on the cell surface, which is relevant for destruction of cancer cells, is via induction of the immune response [18, 70]. Results from several laboratories have demonstrated that cell surface CRT facilitates the phagocytic uptake of apoptotic and cancer cells [71–73]. Clarke and Smyth demonstrated that drug treatments (anthracyclines) caused tumor cell to expose a surface prophagocytic protein, CRT, which induced immunogenic cell death [74]. Additionally, suppression of CRT by siRNA inhibited the anthracycline-induced phagocytosis by dendritic cells and destroyed their immunogenicity in mice [72]. It is becoming clear that surface exposure of CRT is required for phagocytosis on dying tumor cells. CRT expressed on the cell surface is considered as an “eat-me” signal for multiple human cancers, and this prophagocytic function of CRT is disrupted by an antiphagocytic signal CD47 [71]. It has been previously described that an antiphagocytic signal CD47 was increased with high amounts of CRT on cancer cell surfaces to avoid phagocytosis by the immune system [73]. Therefore, interruption of the ability of CD47 by anti-CD47 antibodies might have a therapeutic effect to enhance cancer cell phagocytic uptake [18]. Taken together, these results indicate that CRT-mediated immune mechanisms might be an important strategy for developing new anticancer therapy.

Another interesting question is how this ER chaperone protein gets out of the cell. Several possible mechanisms have been discussed previously [18, 25, 70]. Studies revealed that CRT cotranslocates to the cell surface with ERp57 after anthracycline treatment dictates the immunogenic cell death in preapoptotic cells [70, 75–77]. The exposure pathway of CRT/ERp57 complex is suggested to be triggered by provoking the reactive oxygen species (ROS) or ER stress that activates pancreatic ER kinase (PERK). Activated PERK leads to phosphorylation of the eukaryotic translation inhibition factor eIF2*α*, followed by preapoptotic cleavage of caspase 8 and activation of Bax and Bak [75, 78]. Interestingly, recent studies indicated that ER calcium levels were also involved in CRT translocation to cell surface. Thapsigargin treatment, which leads to ER Ca^2+^ depletion, elevates cell surface expression and secretion of CRT protein [79]. In addition, some chemotherapeutic agents, such as anthracyclines, could also affect the translocation of CRT to the cancer cell surface [80]. CRT expressed on cancer cell surface is important for activation of immune responses. Increasing cell surface CRT exposure may be a potential strategy to develop therapeutics to kill cancer cells.

## 10. Functions of Calreticulin in Regulating Cancer Cell Proliferation

**Figure 2:**
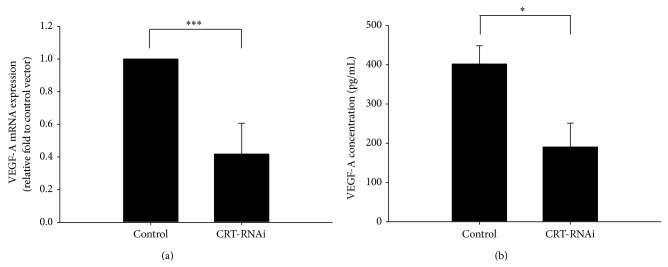
Knockdown of calreticulin suppressed VEGF-A mRNA expression and protein secretion in bladder cancer cell. Details on CRT-knockdown human bladder cancer cell lines (control and CRT-RNAi) were described previously [36]. (a) Real-Time PCR was used to detect VEGF-A mRNA levels in J82 control and CRT-knockdown cells. Total RNA was isolated by the TRIzol reagent following the manufacturer's instructions. Reverse transcription PCR was carried out using ReverTra Ace reverse transcriptase. Real-Time PCR was performed using the iCycler iQ Real-Time detection system (Bio-Rad, Hercules, CA) with the DNA double-strand specific SYBR Green I dye for detection. RNA expression was normalized to the internal control, GAPDH. (b) VEGF-A secretion levels were detected by enzyme-linked immunosorbent assay (ELISA) in conditioned media of J82 control and CRT-knockdown cells. Cells were plated at 5 × 10^5^ cells/well in six-well plates. Conditioned media were collected and analyzed using an ELISA kit specific for human VEGF (BioSource, Camarillo, CA, USA). Statistical differences were compared to the control level (^*^ *P* < 0.05, ^***^ *P* < 0.001).

Cancer formation is characterized by rapid proliferation of mutated cells. Many studies have elucidated that manipulation of CRT levels had profound effects on tumor cell proliferation in diverse types of cancer cells. In pancreatic cells, overexpressed CRT enhanced cell growth; in contrast, knockdown of CRT had the opposite effect on cell growth [48]. In addition, depletion of CRT caused cell cycle arrest at the G0/G1 phase which resulted in significantly suppressed growth rate, colony-formation capacity, and anchorage-independent growth in oral cancer cell [43]. Importantly, Chen et al. have reported that higher levels of CRT promoted cell proliferation and upregulated the proangiogenic factor vascular endothelial growth factor (VEGF) expression in gastric cancer cells [49]. The role of VEGF in regulating angiogenesis has been well documented [81, 82]. As secreted by tumor cells, VEGF binds to specific receptors and activates downstream signal pathways including the mitogen-activated protein kinase (MAPK) and the Ras/extracellular signal-regulated kinase (ERK) which promotes cell proliferation, survival, migration, and angiogenesis [83, 84]. We also found that knockdown of CRT suppressed cell growth in bladder cancer [36]; therefore, we further investigated whether levels of VEGF were also affected by CRT in bladder cancer cells. Results shown in Figure 2 indicated that both expression and secretion levels of VEGF were decreased in CRT-knockdown bladder cancer cells. Although many reports have indicated that CRT has a positive effect on cell growth, other studies provided different viewpoints on this issue. A recent study demonstrated that prostate cancer cells with higher CRT levels produced fewer colonies as well as inhibition of tumor growth both* in vitro* and* in vivo* [85]. Moreover, vasostatin, a fragment of CRT, is considered as an antiangiogenic factor and inhibits VEGF-induced endothelial cell proliferation [86]. Our recent study also demonstrated that CRT upregulates VEGF expression, suppresses cell proliferation, and enhances cell differentiation in neuroblastoma cells [87]. These results strongly suggested that effect of CRT on cell proliferation might depend on cell types.

## 11. Roles of Calreticulin in Neuronal Differentiation in Neuroblastoma

Neuroblastoma (NB) is the most frequently diagnosed malignancy in infancy, with more than 96% of patients diagnosed at the age of <10 years [88, 89]. It is derived from the sympathoadrenal lineage of embryonic neural crest cells [90]. Previous studies suggest that incomplete development and failure of differentiation or apoptosis of neuroblastic cells is critical in its development [91]. Previous studies have shown that NB cells exhibit a capacity of differentiating into mature cells or spontaneous regression by apoptosis [92, 93]. Studies also demonstrated that NB can be forced to differentiate upon the treatment of retinoic acid [94]. On the other hand, NB with better prognosis often express molecular markers indicative of cell differentiation, such as TrkA [95]. Furthermore, the expressions of apoptosis-related genes including p53, Bcl-2, and Bax have been demonstrated in NB and are correlated with favorable prognosis [96]. In some cancers, CRT is found to be upregulated in tumor tissues compared to normal tissue. Conversely, in NB, increased CRT expression is associated with better prognosis and differentiated histologies both* in vitro *and* in vivo* [57, 64]. A CRT knockout (KO) mouse model exhibited embryonic lethality with significant defects in heart, brain, and body wall, suggesting an essential role of CRT in the embryonic development of nervous system [97]. It has been reported that surface CRT is crucial for neurite formation [98]. Moreover, a recent study using PC-12 cells expressing mutant CRT lacking a Ca^2+^-buffering domain (C-domain) suggested that the Ca^2+^-regulating capacity of CRT is essential for NGF-elicited neuronal differentiation [62]. Our recent studies further demonstrated that CRT could suppress cell proliferation and enhance cell differentiation, whereas apoptosis was not altered in NB cells, implying CRT as an important favorable prognostic factor in NB [87]. Besides, we showed that blockage of VEGF signaling could suppress neuronal differentiation in CRT-overexpressed NB cells, suggesting that VEGF-A is involved in CRT-related neuronal differentiation in NB. These results clearly delineate a novel mechanism of CRT during tumorigenesis of NB [87]. These findings also suggest that CRT plays an important role in neuronal differentiation.

## 12. Roles of Calreticulin in Cell Migration and Adhesion

Metastasis is a critical event for cancer progression. This mechanism involves many processes, including cell adhesion, migration, and invasion. Previous studies have revealed that overexpressed CRT contributes to cancer metastasis in gastric, pancreatic, prostate, and ovarian cancers [48, 49, 56, 99]. The possible mechanisms for CRT-mediated cell migration or adhesion have been intensively investigated. One suggested mechanism is that CRT is one of the few cytoplasmic proteins that directly interact with integrin *α*-subunits [15, 17]. In 1995, Coppolino et al. have shown that the interaction between integrin *α*2*β*1 and CRT can be stimulated by integrin activation [100]. They further used the PC-3 prostate cancer cell line as a model to demonstrate that the interaction between integrins and CRT is modulated by phosphorylation and dephosphorylation status [101]. A recent study also reported that integrin-dependent cell adhesion on fibronectin was apparently affected when CRT is overexpressed in epithelial-mesenchymal transition- (EMT-) like cells [102].

Other mechanisms have also proposed that CRT modulates cell adhesion and migration through focal contact dependent manners [34]. This theory is further supported by different levels of CRT affects ECM expressions [39]. Manipulation of CRT expression in mouse L fibroblasts has had a profound effect on fibronectins synthesis. These effects might be due to regulation of c-SRC activity [39]. Cells with higher levels of CRT exhibited increased adhesiveness ability, which is relevant for the calmodulin/calmodulin-dependent kinase II pathway [103]. Moreover, CRT has been reported as a positive regulator for another important focal contact molecule, vinculin. Upregulation of CRT enhanced cell adhesiveness and cell spreading, while knockdown of CRT showed inverse effects in L fibroblast cells [38]. Furthermore, cell surface CRT interacted with thrombospondin to modulate focal adhesion disassembly through the PI3-kinase-dependent pathway [104]. Evidence from these studies suggested that CRT plays a critical role in regulating cell adhesion and migration via various mechanisms.

## 13. New Insight of Calreticulin in Regulation of Integrin Activity

Integrins are heterodimeric transmembrane receptors composed of *α*- and *β*-subunits. They connect extracellular matrix and intracellular cytoskeleton by several cytoplasmic binding proteins to control cell adhesion and migration processes [105]. As we mentioned in previous sections, CRT has been characterized as an intracellular integrin *α*-subunit binding protein and it is essential for integrin-mediated cell adhesion [15, 17]. Meanwhile, little is known about how this mainly ER-resident protein can modulate cell surface receptor functions. According to our latest observation, one critical role of CRT which regulates integrin activation is through modifying *α*1, 2-linkaged glycomic status on *β*1-integrin. Mechanistic investigation demonstrated that CRT controlled the mRNA stability of an important enzyme, fucosyltransferase 1 (FUT1), which catalyzes *α*1, 2-linked fucosylation on *β*1-integrin and subsequently promotes *β*1-integrin activities [106]. These results not only clarify the biological mechanism for CRT regulating integrin functions in cell adhesion process but also provide a new possible strategy for inhibition of cancer metastasis.

## 14. Concluding Remarks

In this review, we summarized the evidences for CRT effects on cancer development. Notably, abnormal CRT levels are highly correlated with pathological outcomes in different types of cancers. Extensive evidences have shown that CRT participates in varieties of cellular functions both inside and outside of ER lumen. The two major functions of CRT are protein chaperoning and Ca^2+^ homeostasis, while mounting evidences indicate that non-ER CRT also plays a crucial role during tumor development. One of the important CRT-mediated mechanisms which regulated cancer cell adhesion is through interaction with integrins. As well as connecting to extracellular matrix, activation of integrins impacts cytoskeletal dynamic by various integrin cytoplasmic-binding proteins [105, 107]. Recently, CRT is known as an integrin *α*-subunit binding protein and it can facilitate *β*1-integrin activation through influencing integrin glycosylation by FUT1 levels. Taking this into consideration, it will be crucial to understand how CRT regulates cell adhesion. It still remained unclear how CRT levels were stimulated in different cancer. Future studies should be required to delineate the possible upstream signal of CRT-related cancer progression, and these results will decipher the roles of CRT in cancer biology.

## Figures and Tables

**Table 1:** Expression of CRT in different cancers.

Cancer	CRT levels^*^	Clinical outcomes	Reference
Oral	Increased	—	[43]
Esophagus	Increased	↓ survival (poor prognosis)	[44]
Breast	Increased	↑ metastasis, ↑ invasion, ↓ survival	[45–47]
Pancreas	Increased	↑ lymph node metastasis, ↑ UICC stage, ↓ survival	[48]
Gastric	Increased	↑ lymph node metastasis, ↑ invasion, ↑ microvessel density, ↓ survival	[49]
Colon	Increased	—	[50]
Bladder	Increased	↑ urinary CRT, ↑ histological grade, ↑ pathological T stage	[51–53]
Prostate	Increased	—	[54]
Vagina	Increased	—	[55]
^ #^Ovarian	Increased	Better response to chemotherapy	[55, 56]
^ #^Neuroblastoma	Increased	↑ differentiation, ↑ survival	[57]

↑: increased; ↓: decreased.

^*^CRT levels in tumor tissue except ovarian carcinoma in effusion fluids.

^#^Higher levels of CRT seem to correlate with good prognosis for the diseases.

## References

[1] Michalak M., Corbett E. F., Mesaeli N., Nakamura K., Opas M. (1999). Calreticulin: one protein, one gene, many functions. *Biochemical Journal*.

[2] Michalak M., Parker J. M. R., Opas M. (2002). Ca^2+^ signaling and calcium binding chaperones of the endoplasmic reticulum. *Cell Calcium*.

[3] Rojiani M. V. (1991). *In vitro* interaction of a polypeptide homologous to human Ro/SS-A antigen (Calreticulin) with a highly conserved amino acid sequence in the cytoplasmic domain of integrin *α* subunits. *Biochemistry*.

[4] Burns K., Duggan B., Atkinson E. A. (1994). Modulation of gene expression by calreticulin binding to the glucocorticoid receptor. *Nature*.

[5] Martin V., Groenendyk J., Steiner S. S. (2006). Identification by mutational analysis of amino acid residues essential in the chaperone function of calreticulin. *The Journal of Biological Chemistry*.

[6] Krause K.-H., Michalak M. (1997). Calreticulin. *Cell*.

[7] Baksh S., Michalak M. (1991). Expression of calreticulin in *Escherichia-coli* and identification of its Ca2+ binding domains. *The Journal of Biological Chemistry*.

[8] Tjoelker L. W., Seyfried C. E., Eddy R. L. (1994). Human, mouse, and rat calnexin cDNA cloning: identification of potential calcium binding motifs and gene localization to human chromosome 5. *Biochemistry*.

[9] Nakamura K., Zuppini A., Arnaudeau S. (2001). Functional specialization of calreticulin domains. *The Journal of Cell Biology*.

[10] Corbett E. F., Oikawa K., Francois P. (1999). Ca^2+^ regulation of interactions between endoplasmic reticulum chaperones. *The Journal of Biological Chemistry*.

[11] Michalak M., Milner R. E., Burns K., Opas M. (1992). Calreticulin. *Biochemical Journal*.

[12] White T. K., Zhu Q., Tanzer M. L. (1995). Cell surface calreticulin is a putative mannoside lectin which triggers mouse melanoma cell spreading. *The Journal of Biological Chemistry*.

[13] Afshar N., Black B. E., Paschal B. M. (2005). Retrotranslocation of the chaperone calreticulin from the endoplasmic reticulum lumen to the cytosol. *Molecular and Cellular Biology*.

[14] Labriola C. A., Conte I. L., Medus M. L., Parodi A. J., Caramelo J. J. (2010). Endoplasmic reticulum calcium regulates the retrotranslocation of *Trypanosoma cruzi* calreticulin to the cytosol. *PLoS ONE*.

[15] Coppolino M. G., Woodside M. J., Demaurex N., Grinstein S., St-Arnaud R., Dedhar S. (1997). Calreticulin is essential for integrin-mediated calcium signalling and cell adhesion. *Nature*.

[16] Dedhar S. (1994). Novel functions for calreticulin: interaction with integrins and modulation of gene expression?. *Trends in Biochemical Sciences*.

[17] Leung-Hagesteijn C. Y., Milankov K., Michalak M., Wilkins J., Dedhar S. (1994). Cell attachment to extracellular matrix substrates is inhibited upon downregulation of expression of calreticulin, an intracellular integrin *α*-subunit-binding protein. *Journal of Cell Science*.

[18] Raghavan M., Wijeyesakere S. J., Peters L. R., del Cid N. (2013). Calreticulin in the immune system: ins and outs. *Trends in Immunology*.

[19] Labriola C., Cazzulo J. J., Parodi A. J. (1999). *Trypanosoma cruzi* calreticulin is a lectin that binds monoglucosylated oligosaccharides but not protein moieties of glycoproteins. *Molecular Biology of the Cell*.

[20] Zapun A., Darby N. J., Tessier D. C., Michalak M., Bergeron J. J. M., Thomas D. Y. (1998). Enhanced catalysis of ribonuclease B folding by the interaction of calnexin or calreticulin with ERp57. *The Journal of Biological Chemistry*.

[21] Vassilakos A., Michalak M., Lehrman M. A., Williams D. B. (1998). Oligosaccharide binding characteristics of the molecular chaperones calnexin and calreticulin. *Biochemistry*.

[22] Spiro R. G., Zhu Q., Bhoyroo V., Söling H.-D. (1996). Definition of the lectin-like properties of the molecular chaperone, calreticulin, and demonstration of its copurification with endomannosidase from rat liver Golgi. *The Journal of Biological Chemistry*.

[23] Pozzan T., Rizzuto R., Volpe P., Meldolesi J. (1994). Molecular and cellular physiology of intracellular calcium stores. *Physiological Reviews*.

[24] Meldolesi J., Pozzan T. (1998). The endoplasmic reticulum Ca^2+^ store: a view from the lumen. *Trends in Biochemical Sciences*.

[25] Michalak M., Groenendyk J., Szabo E., Gold L. I., Opas M. (2009). Calreticulin, a multi-process calcium-buffering chaperone of the endoplasmic reticulum. *Biochemical Journal*.

[26] Araki K., Nagata K. (2011). Protein folding and quality control in the ER. *Cold Spring Harbor Perspectives in Biology*.

[27] Lièvremont J.-P., Rizzuto R., Hendershot L., Meldolesi J. (1997). BiP, a major chaperone protein of the endoplasmic reticulum lumen, plays a direct and important role in the storage of the rapidly exchanging pool of Ca^2+^. *The Journal of Biological Chemistry*.

[28] Bastianutto C., Clementi E., Codazzi F. (1995). Overexpression of calreticulin increases the Ca^2+^ capacity of rapidly exchanging Ca^2+^ stores and reveals aspects of their lumenal microenvironment and function. *The Journal of Cell Biology*.

[29] Mery L., Mesaeli N., Michalak M., Opas M., Lew D. P., Krause K. H. (1996). Overexpression of calreticulin increases intracellular Ca^2+^ storage and decreases store-operated Ca^2+^ influx. *The Journal of Biological Chemistry*.

[30] Lynch J., Guo L., Gelebart P. (2005). Calreticulin signals upstream of calcineurin and MEF2C in a critical Ca^2+^-dependent signaling cascade. *The Journal of Cell Biology*.

[31] Guo L., Nakamura K., Lynch J. (2002). Cardiac-specific expression of calcineurin reverses embryonic lethality in calreticulin-deficient mouse. *The Journal of Biological Chemistry*.

[32] Bibi A., Agarwal N. K., Dihazi G. H. (2011). Calreticulin is crucial for calcium homeostasis mediated adaptation and survival of thick ascending limb of Henle's loop cells under osmotic stress. *The International Journal of Biochemistry & Cell Biology*.

[33] Szabo E., Qiu Y., Baksh S., Michalak M., Opas M. (2008). Calreticulin inhibits commitment to adipocyte differentiation. *The Journal of Cell Biology*.

[34] Villagomez M., Szabo E., Podcheko A., Feng T., Papp S., Opas M. (2009). Calreticulin and focal-contact-dependent adhesion. *Biochemistry and Cell Biology*.

[35] Fadel M. P., Dziak E., Lo C.-M. (1999). Calreticulin affects focal contact-dependent but not close contact-dependent cell-substratum adhesion. *Journal of Biological Chemistry*.

[36] Lu Y. C., Chen C. N., Wang B. (2011). Changes in tumor growth and metastatic capacities of J82 human bladder cancer cells suppressed by down-regulation of calreticulin expression. *The American Journal of Pathology*.

[37] Papp S., Szabo E., Kim H., McCulloch C. A., Opas M. (2008). Kinase-dependent adhesion to fibronectin: regulation by calreticulin. *Experimental Cell Research*.

[38] Opas M., Szewczenko-Pawlikowski M., Jass G. K., Mesaeli N., Michalak M. (1996). Calreticulin modulates cell adhesiveness via regulation of vinculin expression. *The Journal of Cell Biology*.

[39] Papp S., Fadel M. P., Kim H., McCulloch C. A., Opas M. (2007). Calreticulin affects fibronectin-based cell-substratum adhesion via the regulation of c-Src activity. *The Journal of Biological Chemistry*.

[40] Dedhar S., Rennie P. S., Shago M. (1994). Inhibition of nuclear hormone receptor activity by calreticulin. *Nature*.

[41] Coppolino M. G., Dedhar S. (1999). Ligand-specific, transient interaction between integrins and calreticulin during cell adhesion to extracellular matrix proteins is dependent upon phosphorylation/dephosphorylation events. *Biochemical Journal*.

[42] Nickenig G., Michaelsen F., Müller C. (2002). Destabilization of AT_1_ receptor mRNA by calreticulin. *Circulation Research*.

[43] Chiang W.-F., Hwang T.-Z., Hour T.-C. (2013). Calreticulin, an endoplasmic reticulum-resident protein, is highly expressed and essential for cell proliferation and migration in oral squamous cell carcinoma. *Oral Oncology*.

[44] Du X.-L., Hu H., Lin D.-C. (2007). Proteomic profiling of proteins dysregulted in Chinese esophageal squamous cell carcinoma. *Journal of Molecular Medicine (Berl)*.

[45] Lwin Z.-M., Guo C., Salim A. (2010). Clinicopathological significance of calreticulin in breast invasive ductal carcinoma. *Modern Pathology*.

[46] Chahed K., Kabbage M., Ehret-Sabatier L. (2005). Expression of fibrinogen E-fragment and fibrin E-fragment is inhibited in the human infiltrating ductal carcinoma of the breast: the two-dimensional electrophoresis and MALDI-TOF-mass spectrometry analyses. *International Journal of Oncology*.

[47] Bini L., Magi B., Marzocchi B. (1997). Protein expression profiles in human breast ductal carcinoma and histologically normal tissue. *Electrophoresis*.

[48] Sheng W., Chen C., Dong M. (2014). Overexpression of calreticulin contributes to the development and progression of pancreatic cancer. *Journal of Cellular Physiology*.

[49] Chen C.-N., Chang C.-C., Su T.-E. (2009). Identification of calreticulin as a prognosis marker and angiogenic regulator in human gastric cancer. *Annals of Surgical Oncology*.

[50] Alfonso P., Núñez A., Madoz-Gurpide J., Lombardia L., Sánchez L., Casal J. I. (2005). Proteomic expression analysis of colorectal cancer by two-dimensional differential gel electrophoresis. *Proteomics*.

[51] Minami S., Nagashio R., Ueda J. (2014). Detection of tumor-associated antigens in culture supernatants using autoantibodies in sera from patients with bladder cancer. *Biomedical Research*.

[52] Kageyama S., Isono T., Iwaki H. (2004). Identification by proteomic analysis of calreticulin as a marker for bladder cancer and evaluation of the diagnostic accuracy of its detection in urine. *Clinical Chemistry*.

[53] Kageyama S., Isono T., Matsuda S. (2009). Urinary calreticulin in the diagnosis of bladder urothelial carcinoma. *International Journal of Urology*.

[54] Alaiya A., Roblick U., Egevad L. (2000). Polypeptide expression in prostate hyperplasia and prostate adenocarcinoma. *Analytical Cellular Pathology*.

[55] Hellman K., Alaiya A. A., Schedvins K., Steinberg W., Hellström A.-C., Auer G. (2004). Protein expression patterns in primary carcinoma of the vagina. *British Journal of Cancer*.

[56] Vaksman O., Davidson B., Tropé C., Reich R. (2013). Calreticulin expression is reduced in high-grade ovarian serous carcinoma effusions compared with primary tumors and solid metastases. *Human Pathology*.

[57] Hsu W. M., Hsieh F. J., Jeng Y. M. (2005). Calreticulin expression in neuroblastoma—a novel independent prognostic factor. *Annals of Oncology*.

[58] Totary-Jain H., Naveh-Many T., Riahi Y., Kaiser N., Eckel J., Sasson S. (2005). Calreticulin destabilizes glucose transporter-1 mRNA in vascular endothelial and smooth muscle cells under high-glucose conditions. *Circulation Research*.

[59] Qiu Y., Michalak M. (2009). Transcriptional control of the calreticulin gene in health and disease. *The International Journal of Biochemistry & Cell Biology*.

[60] Nguyen T. Q., Capra J. D., Sontheimer R. D. (1996). Calreticulin is transcriptionally upregulated by heat shock, calcium and heavy metals. *Molecular Immunology*.

[61] Vera C., Tapia V., Kohan K. (2012). Nerve growth factor induces the expression of chaperone protein calreticulin in human epithelial ovarian cells. *Hormone and Metabolic Research*.

[62] Shih Y.-Y., Nakagawara A., Lee H. (2012). Calreticulin mediates nerve growth factor-induced neuronal differentiation. *Journal of Molecular Neuroscience*.

[63] Zamanian M., Veerakumarasivam A., Abdullah S., Rosli R. (2013). Calreticulin and cancer. *Pathology & Oncology Research*.

[64] Chang H.-H., Lee H., Hu M.-K. (2010). Notch1 expression predicts an unfavorable prognosis and serves as a therapeutic target of patients with neuroblastoma. *Clinical Cancer Research*.

[65] Klampfl T., Gisslinger H., Harutyunyan A. S. (2013). Somatic mutations of calreticulin in myeloproliferative neoplasms. *The New England Journal of Medicine*.

[66] Nangalia J., Massie C. E., Baxter E. J. (2013). Somatic CALR mutations in myeloproliferative neoplasms with nonmutated JAK2. *The New England Journal of Medicine*.

[67] James C., Ugo V., Le Couédic J.-P. (2005). A unique clonal *JAK2* mutation leading to constitutive signalling causes polycythaemia vera. *Nature*.

[68] Kralovics R., Passamonti F., Buser A. S. (2005). A gain-of-function mutation of JAK2 in myeloproliferative disorders. *The New England Journal of Medicine*.

[69] Sun C., Zhang S., Li J. (2014). Calreticulin gene mutations in myeloproliferative neoplasms without Janus kinase 2 mutations. *Leukemia & Lymphoma*.

[70] Wiersma V. R., Michalak M., Abdullah T. M., Bremer E., Eggleton P. (2015). Mechanisms of translocation of ER chaperones to the cell surface and immunomodulatory roles in cancer and autoimmunity. *Frontiers in Oncology*.

[71] Gardai S. J., McPhillips K. A., Frasch S. C. (2005). Cell-surface calreticulin initiates clearance of viable or apoptotic cells through *trans*-activation of LRP on the phagocyte. *Cell*.

[72] Obeid M., Tesniere A., Ghiringhelli F. (2007). Calreticulin exposure dictates the immunogenicity of cancer cell death. *Nature Medicine*.

[73] Chao M. P., Jaiswal S., Weissman-Tsukamoto R. (2010). Calreticulin is the dominant pro-phagocytic signal on multiple human cancers and is counterbalanced by CD47. *Science Translational Medicine*.

[74] Clarke C., Smyth M. J. (2007). Calreticulin exposure increases cancer immunogenicity. *Nature Biotechnology*.

[75] Panaretakis T., Kepp O., Brockmeier U. (2009). Mechanisms of pre-apoptotic calreticulin exposure in immunogenic cell death. *The EMBO Journal*.

[76] Panaretakis T., Joza N., Modjtahedi N. (2008). The co-translocation of ERp57 and calreticulin determines the immunogenicity of cell death. *Cell Death and Differentiation*.

[77] Obeid M. (2008). ERP57 membrane translocation dictates the immunogenicity of tumor cell death by controlling the membrane translocation of calreticulin. *Journal of Immunology*.

[78] Zitvogel L., Kepp O., Senovilla L., Menger L., Chaput N., Kroemer G. (2010). Immunogenic tumor cell death for optimal anticancer therapy: the calreticulin exposure pathway. *Clinical Cancer Research*.

[79] Peters L. R., Raghavan M. (2011). Endoplasmic reticulum calcium depletion impacts chaperone secretion, innate immunity, and phagocytic uptake of cells. *Journal of Immunology*.

[80] Tufi R., Panaretakis T., Bianchi K. (2008). Reduction of endoplasmic reticulum Ca^2+^ levels favors plasma membrane surface exposure of calreticulin. *Cell Death & Differentiation*.

[81] Sene A., Chin-Yee D., Apte R. S. (2015). Seeing through VEGF: innate and adaptive immunity in pathological angiogenesis in the eye. *Trends in Molecular Medicine*.

[82] Senger D. R., van de Water L., Brown L. F. (1993). Vascular permeability factor (VPF, VEGF) in tumor biology. *Cancer and Metastasis Reviews*.

[83] Liang X., Xu F., Li X., Ma C., Zhang Y., Xu W. (2014). VEGF signal system: the application of antiangiogenesis. *Current Medicinal Chemistry*.

[84] Roberts E., Cossigny D. A. F., Quan G. M. Y. (2013). The role of vascular endothelial growth factor in metastatic prostate cancer to the skeleton. *Prostate Cancer*.

[85] Alur M., Nguyen M. M., Eggener S. E. (2009). Suppressive roles of calreticulin in prostate cancer growth and metastasis. *The American Journal of Pathology*.

[86] Shu Q., Li W., Li H., Sun G. (2014). Vasostatin inhibits VEGF-induced endothelial cell proliferation, tube formation and induces cell apoptosis under oxygen deprivation. *International Journal of Molecular Sciences*.

[87] Weng W.-C., Lin K.-H., Wu P.-Y. (2014). Calreticulin regulates VEGF-A in neuroblastoma cells. *Molecular Neurobiology*.

[88] Brodeur G. M. (2003). Neuroblastoma: biological insights into a clinical enigma. *Nature Reviews Cancer*.

[89] Chang H.-H., Hsu W.-M. (2010). Neuroblastoma—a model disease for childhood cancer. *Journal of the Formosan Medical Association*.

[90] Maris J. M., Hogarty M. D., Bagatell R., Cohn S. L. (2007). Neuroblastoma. *The Lancet*.

[91] Israel M. A. (1993). Disordered differentiation as a target for novel approaches to the treatment of neuroblastoma. *Cancer*.

[92] Ijiri R., Tanaka Y., Kato K. (2000). Clinicopathologic study of mass-screened neuroblastoma with special emphasis on untreated observed cases: a possible histologic clue to tumor regression. *The American Journal of Surgical Pathology*.

[93] Nishihira H., Toyoda Y., Tanaka Y. (2000). Natural course of neuroblastoma detected by mass screening: a 5-year prospective study at a single institution. *Journal of Clinical Oncology*.

[94] Sidell N., Altman A., Haussler M. R., Seeger R. C. (1983). Effects of retinoic acid (RA) on the growth and phenotypic expression of several human neuroblastoma cell lines. *Experimental Cell Research*.

[95] Nakagawara A., Arima-Nakagawara M., Scavarda N. J., Azar C. G., Cantor A. B., Brodeur G. M. (1993). Association between high levels of expression of the TRK gene and favorable outcome in human neuroblastoma. *The New England Journal of Medicine*.

[96] Hoehner J. C., Gestblom C., Olsen L., Påhlman S. (1997). Spatial association of apoptosis-related gene expression and cellular death in clinical neuroblastoma. *British Journal of Cancer*.

[97] Rauch F., Prud'Homme J., Arabian A., Dedhar S., St-Arnaud R. (2000). Heart, brain, and body wall defects in mice lacking calreticulin. *Experimental Cell Research*.

[98] Xiao G., Chung T.-F., Pyun H. Y., Fine R. E., Johnson R. J. (1999). KDEL proteins are found on the surface of NG108-15 cells. *Molecular Brain Research*.

[99] Wu M., Bai X., Xu G. (2007). Proteome analysis of human androgen-independent prostate cancer cell lines: variable metastatic potentials correlated with vimentin expression. *Proteomics*.

[100] Coppolino M., Leung-Hagesteijn C., Dedhar S., Wilkins J. (1995). Inducible Interaction of Integrin *α*
_2_
*β*
_1_ with calreticulin: dependence on the activation state of the integrin. *Journal of Biological Chemistry*.

[101] Coppolino M. G., Dedhar S. (1999). Ligand-specific, transient interaction between integrins and calreticulin during cell adhesion to extracellular matrix proteins is dependent upon phosphorylation/dephosphorylation events. *Biochemical Journal*.

[102] Ihara Y., Inai Y., Ikezaki M. (2011). Alteration of integrin-dependent adhesion and signaling in EMT-like MDCK cells established through overexpression of calreticulin. *Journal of Cellular Biochemistry*.

[103] Szabo E., Papp S., Opas M. (2007). Differential calreticulin expression affects focal contacts via the calmodulin/CaMK II pathway. *Journal of Cellular Physiology*.

[104] Goicoechea S., Orr A. W., Pallero M. A., Eggleton P., Murphy-Ullrich J. E. (2000). Thrombospondin mediates focal adhesion disassembly through interactions with cell surface calreticulin. *The Journal of Biological Chemistry*.

[105] Morse E. M., Brahme N. N., Calderwood D. A. (2014). Integrin cytoplasmic tail interactions. *Biochemistry*.

[106] Lu Y.-C., Chen C.-N., Chu C.-Y. (2014). Calreticulin activates *β*1 integrin via fucosylation by fucosyltransferase 1 in J82 human bladder cancer cells. *The Biochemical Journal*.

[107] Ciobanasu C., Faivre B., le Clainche C. (2013). Integrating actin dynamics, mechanotransduction and integrin activation: the multiple functions of actin binding proteins in focal adhesions. *European Journal of Cell Biology*.

